# Mucosal IFNγ production and potential role in protection in *Escherichia coli* O157:H7 vaccinated and challenged cattle

**DOI:** 10.1038/s41598-021-89113-7

**Published:** 2021-05-07

**Authors:** Robert G. Schaut, Mitchell V. Palmer, Paola M. Boggiatto, Indira T. Kudva, Crystal L. Loving, Vijay K. Sharma

**Affiliations:** 1 USDA-ARS, National Animal Disease Center, 1920 Dayton Avenue, P.O. Box 70, Ames, IA 50010 USA; 2 Food Safety and Enteric Pathogens Research Unit, Ames, IA USA; 3 Oak Ridge Institute for Science and Education (ORISE), ARS Research Participation Program, Oak Ridge, TN USA; 4 Infectious Bacterial Diseases Research Unit, Ames, IA USA; 5 Elanco, 2500 Innovation Way, Greenfield, IN 46140 USA

**Keywords:** Immunology, Microbiology

## Abstract

Shiga-toxin producing *Escherichia coli* O157:H7 (O157)-based vaccines can provide a potential intervention strategy to limit foodborne zoonotic transmission of O157. While the peripheral antibody response to O157 vaccination has been characterized, O157-specific cellular immunity at the rectoanal junction (RAJ), a preferred site for O157 colonization, remains poorly described. Vaccine induced mucosal O157-specific antibodies likely provide some protection, cellular immune responses at the RAJ may also play a role in protection. Distinct lymphoid follicles were increased in the RAJ of vaccinated/challenged animals. Additionally, increased numbers of interferon (IFN)γ-producing cells and γδ + T cells were detected in the follicular region of the RAJ of vaccinated/challenged animals. Likewise, adjuvanted-vaccine formulation is critical in immunogenicity of the O157 parenteral vaccine. Local T cell produced IFNγ may impact epithelial cells, subsequently limiting O157 adherence, which was demonstrated using in vitro attachment assays with bovine epithelial cells. Thus, distinct immune changes induced at the mucosa of vaccinated and challenged animals provide insight of mechanisms associated with limiting O157 fecal shedding. Enhancing mucosal immunity may be critical in the further development of efficacious vaccines for controlling O157 in ruminants and thus limiting O157 transmission to humans.

## Introduction

Shiga toxin-producing *Escherichia coli* O157:H7 (O157) is a causative agent of bloody diarrhea, or hemorrhagic colitis, which in very young, elderly, and people with underlying immune disorders could lead to kidney failure due to the development of hemorrhagic uremic syndrome (HUS)^1^. The development of HUS is a potentially life-threatening condition and requires patient hospitalization^2^. During O157-mediated disease, non-ambulatory care is generally supportive (intra-venous fluids, dialysis in cases of hemolytic uremic syndrome) as antibiotics are contraindicated^3^. In particular, human disease is acquired by zoonotic transmission from cattle via the consumption of contaminated meat, vegetables or water^4–10^. Although safe food handling practices can help to minimize disease transmission of O157, targeting the reservoir host animal (cattle) may be an important facet in reducing O157 disease incidence in humans. An economical method of host animal targeting is by vaccination, which can prevent or reduce colonization and fecal shedding after O157 exposure of these animals.

In a previous study, we demonstrated a 50% reduction in fecal shedding of O157 from experimentally inoculated animals if they were vaccinated with a vaccine formulation containing an inactivated *hha* deletion mutant strain of O157 overexpressing the type 3 secretion system proteins (T3SP)^11^. In a subsequent study, we demonstrated that the addition of an adjuvant to the vaccine formulation was important since animals vaccinated with the adjuvanted vaccine showed both a reduction in the duration of fecal shedding (mean 14 days) and amount of O157 shed in feces (10^5^ CFU/gram feces) compared to longer duration of fecal shedding (mean 30 days) and higher amount of O157 shed in feces (10^7^ CFU/gram feces) of sham vaccinated cattle^12^. Although our group and others have identified immunological indicators associated with reduced shedding, such as O157-specific IgG and IgA^12–17^ or peripheral O157 specific T cells responses^18^, tissue specific immunological responses to O157 vaccination regimens are not as clearly defined.

O157 can colonize and induce microscopic attaching and effacing (A/E) lesions at the recto-anal junction (RAJ) in neonatal and weaned-fasted cattle^19–23^. However, minimal pathology is detected in conventional cattle. Mucosal O157-specific IgA in tissue homogenates as well as an increase in granulocytes suggests that the immune system may actively respond to O157 colonization, however impact on shedding may be minimal^24^. Others have suggested that O157 acts as a commensal at the colonization site^25,26^ by subverting an immune response through the expression of pathogenic traits to suppress immunity^27^. One study suggests that vaccination supported tissue-specific inflammatory (localized to the rectal-lymph node) as well as CD4^+^ T cell responses that may be contributing to a reduction in fecal shedding^28^. Transcriptional analysis of the terminal rectal tissues also supports the findings where vaccination elicited inflammation, which may contribute to an adaptive immune response that limits O157 colonization or fecal shedding^29^.

The aim of this study was to partially identify T cell cytokine-bias, including how these cytokines may impact O157 tissue adherence at the RAJ, in response to a vaccination scheme that resulted in 50% reduced fecal shedding of O157 given orally to the vaccinated cattle^12^. Data from animals vaccinated against O157 with the adjuvanted-vaccine suggested that a decrease in circulating CD8 + γδ T cells and cell mediated responses measured in the periphery^18^ suggesting further investigation into cell populations, especially at the site of the RAJ. We hypothesized that there would be changes in lymphocyte populations, specifically γδ T cells, within the RAJ and these cell populations skew toward interferon-γ biased responses in response to successful O157 vaccination scheme.

## Results

### Increased lymphocytes in RAJ of O157-vaccinated and challenged animals

**Figure 1:**
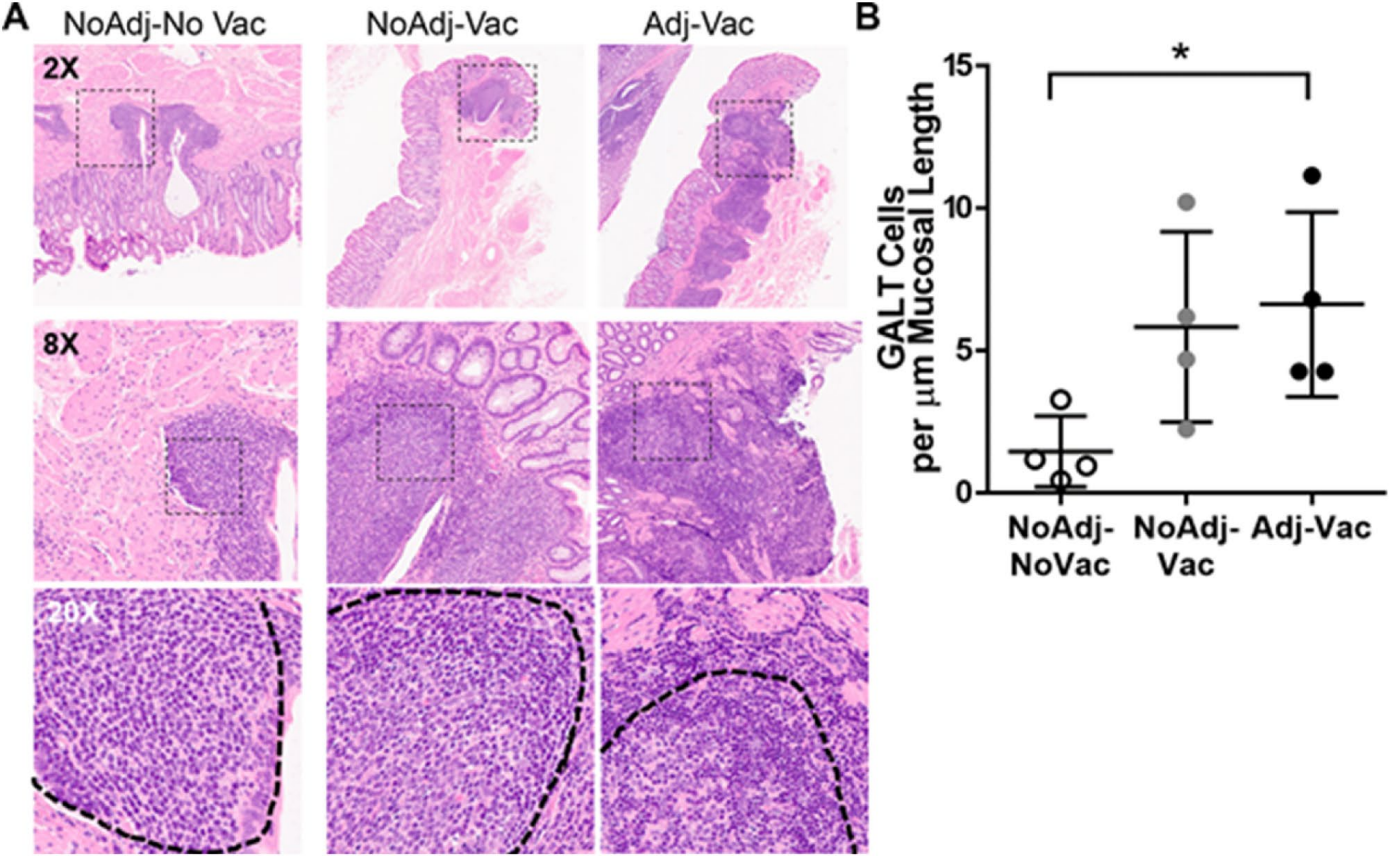
Increased lymphoid structures in rectoanal junction of vaccinated and challenged cattle. Histologic examination of recto-anal junction (RAJ) sections revealed increased gut-associated lymphoid tissue (GALT). Representative slides of RAJ collected from non-vaccinated (NoAdj-NoVac; left panels), non-adjuvanted-Δ*hha E. coli*-vaccinated (NoAdj-Vac) (middle panels) and oil-in water adjuvanted-Δ*hha E. coli*-vaccinated (Adj-Vac) (right panels) are shown (**A**). Panels increase in magnification from top (2 ×), middle (8 ×) and bottom (20 ×) panels. Hashed box indicates section of magnification shown in bottom panel. Dashed line in bottom row represents follicle boundary. (**B**) Quantification of GALT cells per um of mucosal length. Open circles represent NoAdj-NoVac, grey circles represent NoAdj-Vac, and black circles represent Adj-Vac. Three replicate tissue sections from each animal were analyzed and averaged for single data point for each animal. *p < 0.05 by one-way ANOVA with Tukey’s post-test.

To identify any changes in cellularity of the recto-anal junction (RAJ), sections of the RAJ were collected at the end of the study (day 72) for histological analysis. The follicular and squamous regions of the RAJ were sectioned, H&E stained and analyzed utilizing a digital analysis system to measure GALT hyperplasia. Lymphoid follicle formation associated with GALT hyperplasia was observed within the submucosa, at times extending superficially to the mucosal surface in the follicular RAJ isolated from adjuvant-vaccinated-challenged (Adj-Vac) animals (Fig. 1A, right panels). There was a modest increase in lymphoid follicles in the RAJ tissues collected from non-adjuvanted-vaccinated-challenged (NoAdj-Vac) animals (Fig. 1A, middle panels), whereas the challenge only animals (NoAdj-NoVac) demonstrated the lowest number of follicles (Fig. 1A, left panels). RAJ-tissue follicles were significantly (p < 0.01) greater when compared to the RAJ sections from NoAdj-NoVac and Adj-Vac animals (Fig. 1B).

### Increased abundance of IFNγ-positive and γδ T cells in follicular but not squamous region of RAJ

**Figure 2:**
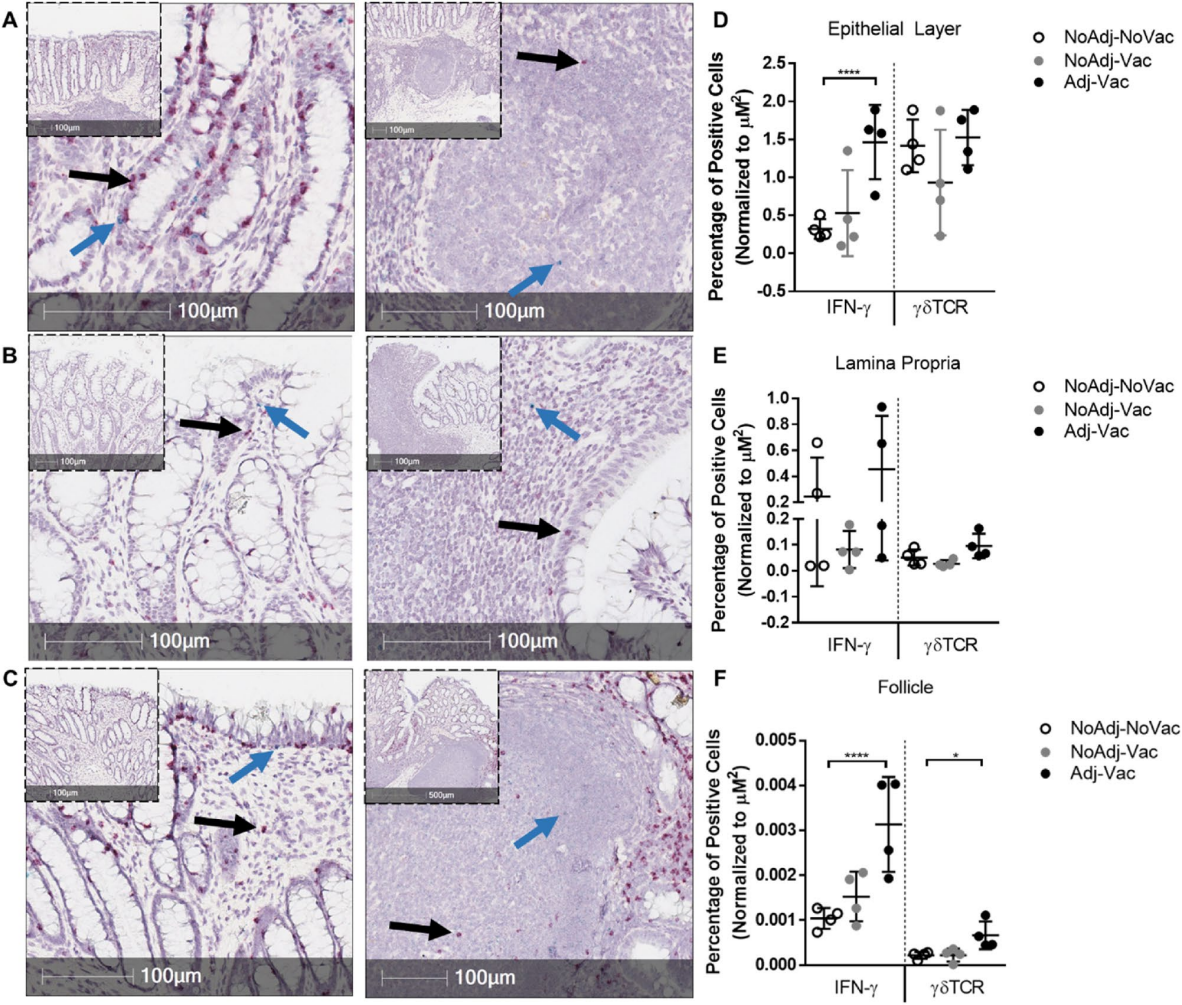
RNA in situ hybridization of IFNG and TRDC transcripts. Follicular recto-anal junction (RAJ) isolated from a representative animal which received no adjuvant or vaccine strain (NoAdj-NoVac) (**A**) depicting mucosal epithelium (left panel) and submucosa-follicle (right panel). Follicular RAJ isolated from a representative animal which received non-adjuvanted-Δ*hha*-*E. coli* vaccine (NoAdj-Vac) (**B**) depicting mucosal epithelium (left panel) and submucosa-follicle (right panel). Follicular RAJ isolated from a representative animal which received adjuvanted-Δ*hha*-*E. coli* vaccine (Adj-Vac) (**C**) depicting mucosal epithelium (left panel) and submucosa-follicle (right panel). Graphical representation of γδTCR^+^ (right) and IFNγ^+^ (left) from mucosal epithelium (**D**), submucosa lumen (**E**) and submucosa-follicle (**F**). On inset panels indicated in (**A**–**C**), an approximate 5 × magnification section is represented whereas larger panels are approximately 20 × magnification of the inset. Black arrows indicate γδ T cell staining (specifically, the T-cell receptor, delta chain RNA) and blue arrows indicate IFNγ (interferon RNA) staining. Open circles indicate NoAdj-NoVac, grey circles represent NoAdj-Vac, and black circles represent Adj-Vac group. Each symbol represents an individual animal. N = 4 per group. Bars =  ± SD. Bar data represents percentage of cells measured by RNA staining. One-way ANOVA with Tukey’s post-test was utilized for statistical analysis. ****p < 0.001, *p < 0.05.

With the evidence of increased numbers of lymphocytes in the RAJ (Fig. 1) of the Adj-Vac group, and data from previous studies^18^ suggestive of an important role of circulating lymphocytes, specifically γδ^+^ T cells, characterization of the lymphocytes in the RAJ of the various experimental groups can lend some insight into the effect of vaccination on reduced fecal shedding of O157. Thus, we examined IFNγ^+^ and γδ^+^ T cells in the follicular and squamous regions of RAJ. RNA in situ hybrization analysis of the follicular and squamous regions for expression of IFNG (interferon-gamma gene) and TRDC (T cell receptor, delta chain) mRNA indicated increased abundance of IFNG and TRDC expressing cells in the Adj-Vac group (Fig. 2C, left panels, arrows) although considerable numbers of IFNG and TRDC positive cells were detected in the NoAdj-NoVac group as well (Fig. 2A, left panels, arrows). On the other hand, the NoAdj-Vac group did not have increased IFNG expressing cells relative to the mock vaccinated group (Fig. 2B, left panel). It is possible that the TRDC expressing cells, some of which were positive for IFNG gene expression (data not shown), contributed toward an inflammatory environment within the follicular region of the RAJ of the vaccinated animals (Fig. 2D–F).

**Figure 3:**
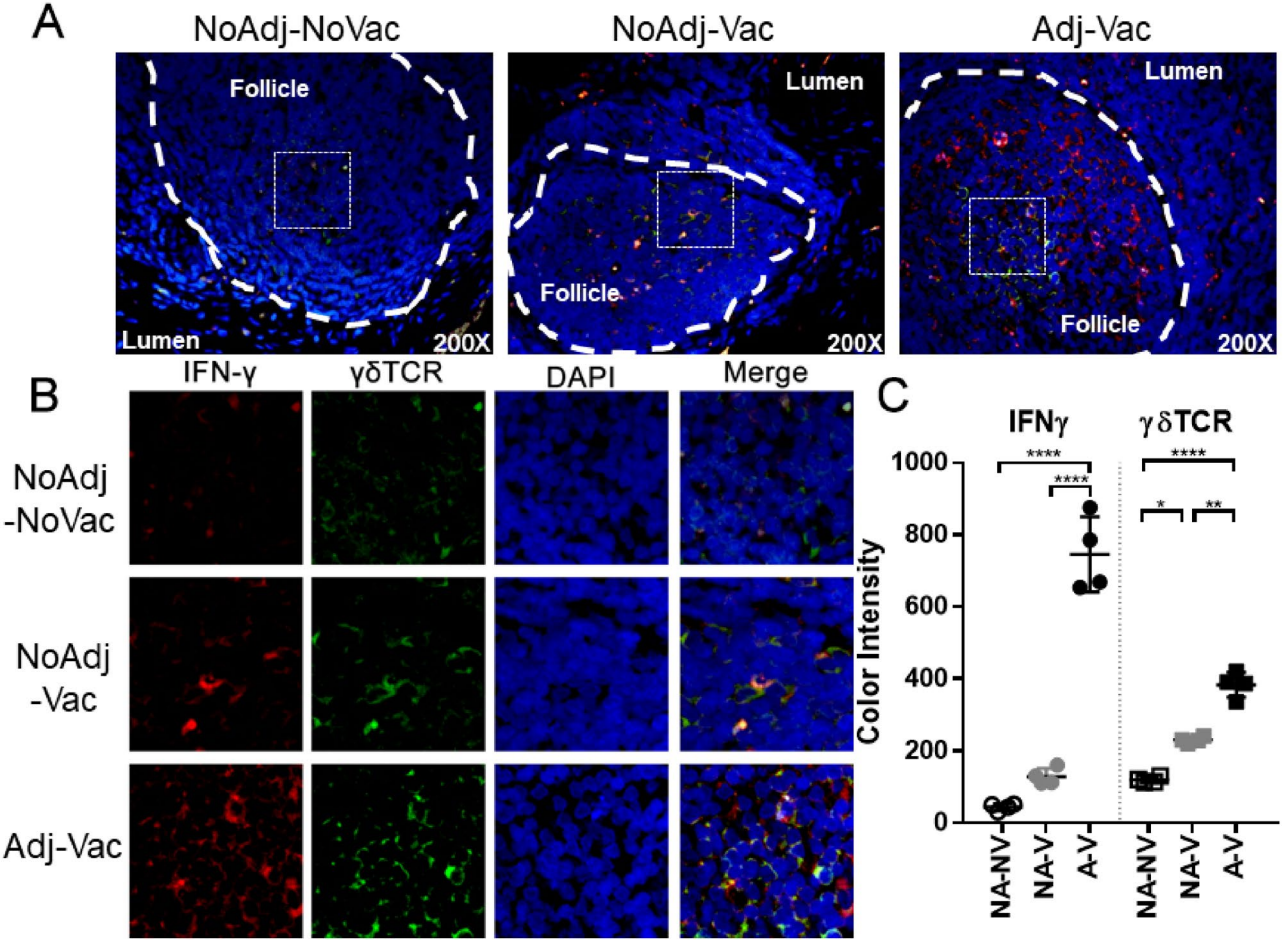
Increased abundance of IFNγ-positive cells in RAJ of cattle receiving adjuvanted vaccine. Characterization of cells within follicles of the recto-anal junction by immunofluorescent staining for either γδ TCR or IFNγ (**A**, **B**). Follicular region of RAJ was sectioned and labeled with florescent antibodies directed toward IFNγ (red) or γδTCR (green) and stained with nuclear stain DAPI (blue). Dashed lines indicate follicle boundary and hashed box indicate magnified section shown in (**B**) with representative section as indicated from an animal in non-adjuvanted-non-vaccinated (NoAdj-NoVac; NA-NV), non-adjuvanted-vaccine (NoAdj-Vac; NA-V), or adjuvanted-vaccine (Adj-Vac; A-V) groups. (**C**) Graphical representation of florescent data from indicated groups. Bars are ± SD. ****p < 0.0001, **p < 0.01, *p < 0.05 one-way ANOVA with Tukey’s post-test.

The squamous region of the RAJ did have some IFNG or TRDC expressing (positive for RNA staining) cells (Supplementary Fig. 1A) but the abundance of IFNG and TRDC positive cells was not significantly different between the groups (Supplementary Fig. 1B, bars represent percentage of cells measured by RNA staining, ± SD) and was far reduced when compared to the follicular region. Hence, sections of follicular RAJ were stained for IFNγ (red) and γδTCR (green) protein (Fig. 3A). Within the lymphoid follicles, larger areas of both IFNγ and γδTCR staining were observed in the Adj-Vac groups compared to the NoAdj-Vac and NoAdj-NoVac animals (Fig. 3B). As shown in Fig. 3C, relative amount of IFNֱγ (red) and γδTCR (green) signal detected was significantly higher in tissue sections from Adj-Vac animal group compared to the NonAdj-NonVac (p < 0.0001) and NonAdj-Vac animals (*p* < 0.0001 for IFNγ and *p* < 0.001 for γδTCR). (Fig. 3C). Furthermore, when examining the mesenteric lymph node and spleen, more IFN-γ producing lymphocytes were detected in the Adj-Vac animals compared to the other experimental groups (Supplemental Fig. 2).

### Altered expression of immune genes in follicular RAJ of O157 vaccinated animals

**Figure 4:**
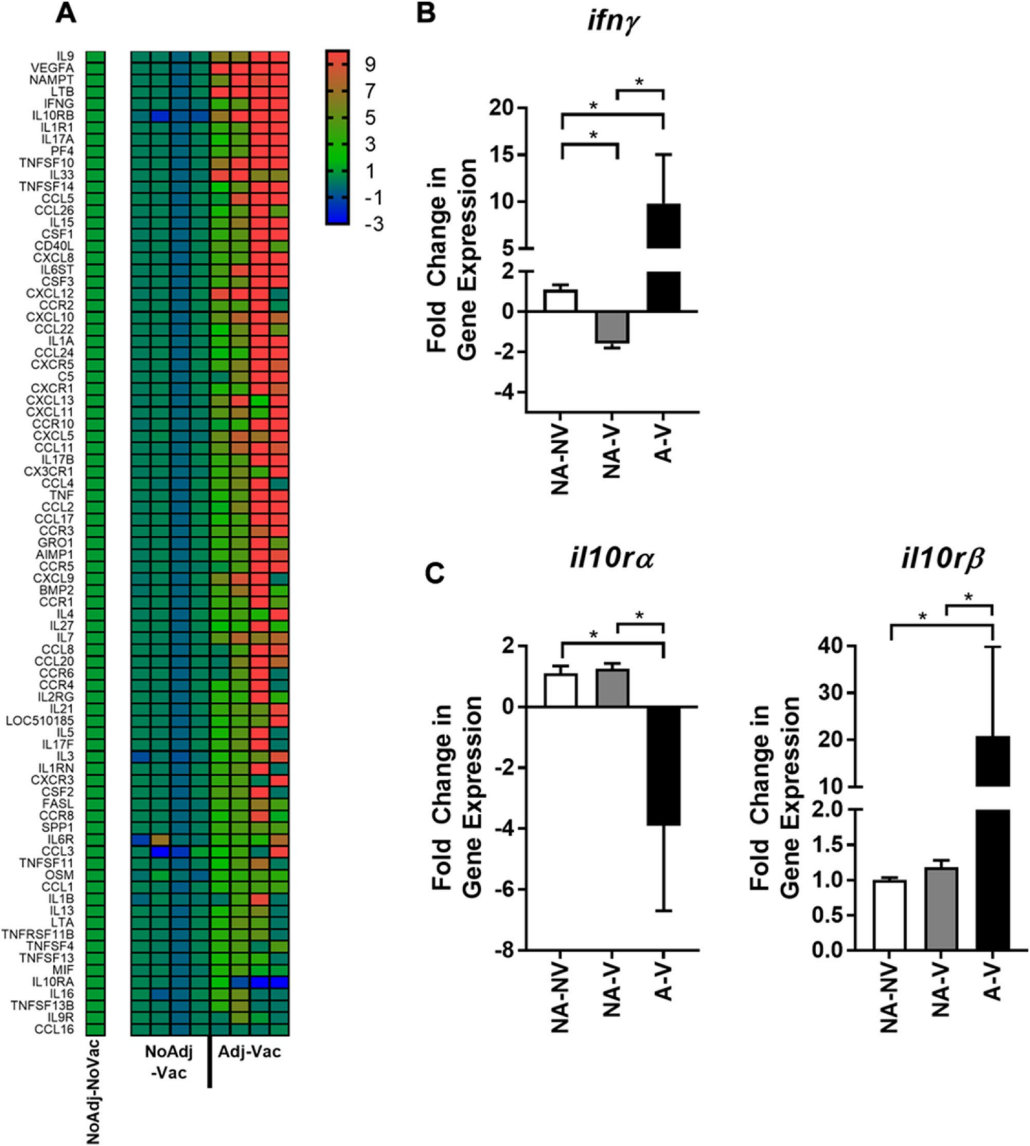
Transcriptional analysis of cells from follicular region of recto-anal junction. Transcriptomic analysis was performed utilizing single-step reverse-transcription quantitative PCR (RT-qPCR) reactions for targets indicated. (**A**) Heat map displays fold change of target genes in non-adjuvanted-vaccinated (NoAdj-Vac; NA-V) and adjuvanted-vaccinated (Adj-Vac; A-V) animals relative to non-vaccinated-non-adjuvanted (NoAdj-NoVac; NA-NV) animals utilizing the 2^−ΔΔCT^ method. Values were Log_2_ transformed to reflect negative fold-change. Red values indicate increased transcript levels, green indicates no change, and blue indicates decreased levels when compared to levels in cells from NoAdj-NoVac group. Each column represents a single animal in the listed treatment group. Values were normalized to 3 housekeeping genes to account for variation in RNA loading. Changes in IFNγ (**B**) and IL10RA and IL10RB (**C**) are shown to highlight a few specific genes. N = 4 per group. Bars =  ± SD. One-way ANOVA with Tukey’s post-test was utilized for statistical analysis. *p < 0.05.

To further understand immune response in follicular RAJ region, transcriptional analysis via reverse transcription quantitative PCR (RT-qPCR) was performed to evaluate relative expression of inflammatory meditators including those associated with IFNγ-biased responses. RNA isolated from follicular RAJ of NoAdj-Vac animals had decreased or unchanged expression (blue or green, respectively) of many genes when compared to challenge only controls (NoAdj-NoVac; Fig. 4A, left columns, Supplementary Table S1). Conversely, most genes analyzed were increased (red) in the follicular RAJ of Adj-Vac animals (Fig. 4A, right columns) when compared to NoAdj-NoVac group. For example, as highlighted in Fig. 4B, the IFNγ gene expression was increased in the Adj-Vac animals and decreased in the NoAdj-Vac animals compared to NoAdj-NoVac control animals. The IL-10 receptor is a complex of alpha receptor (IL-10RA) and beta receptor (IL-10RB); the NoAdj-Vac group was not statistically different than the NonAdj-NoVac group for IL10R gene expression whereas a decrease in IL-10RA expression and increase in IL10RB was detected in the Adj-Vac group (Fig. 4A, C).

### IFNγ treatment reduced O157 adherence to HEp-2 cells and bovine intestinal epithelial cells (BIEC)

**Figure 5:**
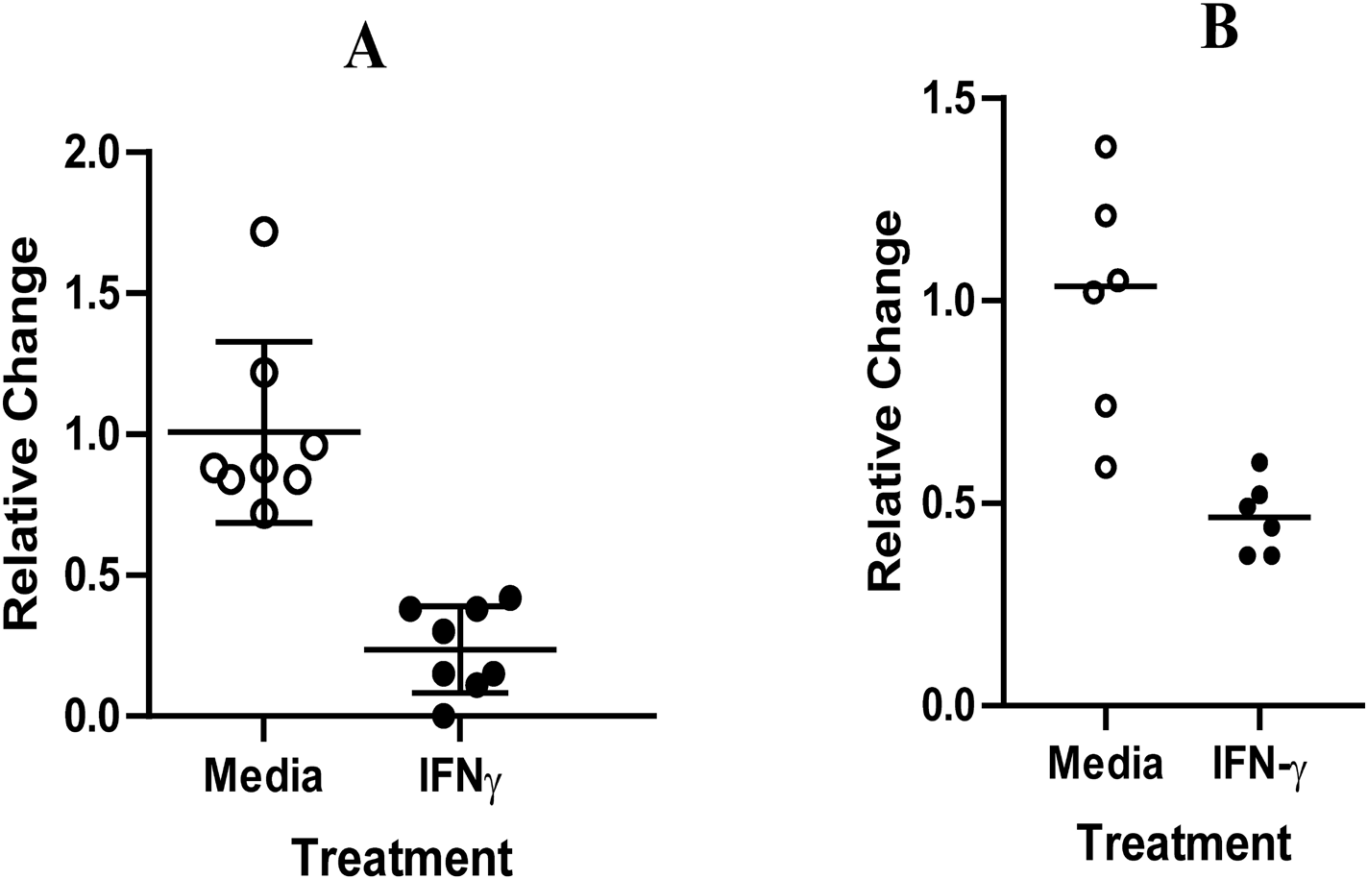
Treatment of epithelial cells with IFNγ limits attachment of O157. HEp-2 cells (**A**) and bovine intestinal epithelial cells (BIEC) (**B**) were either untreated (Medium only, open circles) or pretreated with recombinant bovine IFNγ (IFNγ, black circles) and subsequent O157 attachment assessed as described in Materials and Methods. (**A**) Results presented are the average of two independent assays, and each assay included four wells. Bars are ± SD. **p < 0.01 determined by One-way ANOVA with Dunnet posttest, (**B**) The data is expressed relative to average attached O157 in media-only wells. Data is representative of two biological experiment (n = 2) and three technical replicates.

IFNγ-producing cells were detected within the RAJ lymphoid follicles and its role in potentially limiting shedding in O157-vaccinated animals is unclear. To begin to explore a role of IFNγ in limiting O157 adherence to epithelial cells, we assessed the impact of recombinant IFNγ treatment of two different cell lines on adherence of O157 on epithelial cells. Recombinant bovine IFNγ was added to HEp-2 cells followed by inoculation of O157. Adherent O157 were enumerated. HEp-2 cells stimulated with IFNγ showed significantly (*p* < 0.05) reduced O157 adherence (Fig. 5A, black lines with black filled circles). While Hep-2 cells are often used as a proxy for O157 attachment to epithelial cells, the O157 proteins involved in attachment to HEp-2 versus bovine cells may be different^30^. To examine the ability of IFNγ to limit O157 attachment to bovine cells, a bovine intestinal epithelial cell line (BIEC-c4)^31^ was used in attachment assays. The addition of recombinant bovine IFNγ to BIEC-c4 cells limited subsequent attachment of O157 to the BIEC cells by about 40% (Fig. 5B). Thus, locally produced IFNγ may impact RAJ epithelial cells to limit attachment, which may contribute to reduced fecal shedding of O157 by vaccinated animals.

## Discussion

Bovine responses to vaccination against Shiga-toxin producing *E. coli* O157:H7 (O157) have been described previously through serology^11,12^, presence of anti-O157 antibodies in feces of vaccinated animals^18^, and induction of peripheral T-cell responses^18^. A few studies have examined the effects of O157 challenge on transcriptional changes within the recto-anal junction (RAJ)^27,29,32^, changes in cellular populations within the periphery^32^, the Peyer’s patch^27^ or the rectal lymph node^28^. Here, a close examination of the tissue changes which occurred within the follicular region of the RAJ following vaccination and O157 challenge of cattle are described. As antibodies or peripheral responses may only serve as a partial prognosticative indicator of vaccine efficacy, it is likely that site-specific responses contribute to reduced colonization and shedding of O157 from the vaccinated ruminant animal.

O157 bacteria can transiently colonize the intestinal tract of cattle for a few days by adhering to sites within the rumen, reticulum, and spiral colon^33^; however, O157 preferential colonization at the RAJ could result in a longer duration of shedding lasting one to two months^30,34–36^. O157 colonization is typically associated with subclinical responses within cattle such as microscopic lesion development at RAJ^37,38^. However, other effects such as an approximately 20% increase in site-specific CD4^+^ T cells^32^ and a mild granulocytic infiltration within the lamina propria of the rectum^24^ may be associated with O157 colonization. As there were lymphocytic aggregates identified in RAJ of each of the experimental groups within our study, it is likely that O157 by itself may be contributing to an increase or change in the cellular profile of the RAJ. Furthermore, vaccination may be driving additional changes within the cellular makeup of the RAJ either through an increase in the total number of leukocytes (Fig. 1) or the type of lymphocytes present within the tissue (Fig. 2). Future studies which examine a time course of cellular infiltration or expansion either after vaccination, O157 challenge, or vaccination and challenge would be valuable in identifying important lymphocyte populations leading to reduced fecal shedding. We have demonstrated in a previous study that animals injected with the adjuvanted only preparation had no effect on *E. coli* O157:H7 shedding and failed to induce circulating IgG response^12^. However, additional immunological assessment of adjuvant-only treatments could be performed in the future study to help delineate the usefulness of vaccine formulation within the context of adjuvant-directed, cell-specific host responses.

IFNγ can be an important mediator of macrophage activation or changes within the mucosal epithelium to prevent bacterial infiltration^39^. Here, an increase of follicle formation within the mucosa, which was accompanied by an increase in IFNγ-producing cells, associated to previous findings showing that vaccination of animals with the adjuvanted vaccine formulation was important in reducing the magnitude and duration of fecal shedding of O157^12^. Similar to these current findings, others have demonstrated that the fecal shedding kinetics of a low-shedding *E. coli* strain of O157:H7 compared to a high shedding strain did correlate with increased IFNγ and other Th1 skewing transcripts within the low-shedding group^32^. Here, the primary focus included IFNγ and IL-10 receptor gene expression as these genes relate to the protein and cellular expression previously documented by our group^18^, however many other inflammatory genes were analyzed and found to be significantly differential between the experimental groups (Fig. 4, Supplementary Table S1). Further studies to identify biological significance of these findings are necessary.

Outside of intestinal aspects of IFNγ, when dairy cows were pre-treated with recombinant IFNγ and challenged intra-mammary with *E. coli*, animals demonstrated reduced duration of infection and lower clinical scores of mastitis compared to non-treated animals^40^. Effects of IFNγ on *E. coli* strains outside of ruminant hosts have also been explored. Administration of recombinant IFNγ to avian pathogenic *E. coli*-challenged chickens increased the phagocytic ability of macrophages coupled with an overall increase in MHCII expression on leukocytes within the periphery, suggesting a possible therapeutic use of IFNγ under certain circumstances^41^. Similarly, IFNγ receptor expression may also play a role in the development of a favorable environment for *E. coli* colonization. For example, enterohemorrhagic *E. coli* (EHEC) infection in a murine model led to an altered IFNγ-receptor expression and suppression of IFNγ-biased signaling, suggesting that reduced effects of IFNγ may be an important part of *E. coli* pathology^42^. Overall, it is likely that IFNγ may play a role in ameliorating *E. coli* colonization, infection or recovery; future studies examining the site-specific responses of IFNγ are necessary.

The localization of specific subsets of immune cells within the intestinal tract of the ruminant host after O157 challenge may be important in driving reduced O157 fecal shedding. Previously, our finding from the animals within this reported study have suggested that antigen-specific γδ^+^ T cells can produce IFNγ to a greater extent in response to O157 than autologous CD4^+^ T cells after vaccination^18^. In the current study, γδ T cells were found in the follicular RAJ, although the association between O157 vaccination-driven responses of antigen-specific γδ T cells and the ruminant host are relatively unexplored. As cattle have an extensive gastrointestinal tract, γδ T cells are believed to be essential in the regulation of immune responses of the intestinal and respiratory mucosa^43^. Based on cell surface phenotyping, two distinct subsets of γδ T cells, namely CD8^−^ γδ TCR^+^ and CD8^+^ γδ TCR^+^ cells have been identified in cattle. Although both subsets have overlapping functionality, it has been demonstrated that γδ TCR^+^ CD8^+^ cells are sensitive to IFNγ and promote cellular proliferation in conjunction with IL-2R signaling^44^. It is possible that the γδ TCR^+^ CD8^+^ subset of cells may play a greater role in IFNγ-biased inflammatory response compared to their γδ TCR^+^ CD8^−^ counterparts. As γδ T cells are important mucosal-associated cells capable of producing IFNγ, it would be likely that their role in the IFNγ-biased immune responses directed toward O157 would be essential in the reduction of fecal shedding of O157. However, more studies are necessary to determine the role of γδ T cells and their cellular subsets in response to O157 including investigation into broader role of adjuvants in an O157-vaccine setting. Likewise, additional studies to describe CD4^+^, CD8^+^ and other IFNγ-producing cells (i.e. NK cells) are necessary to glean a wholistic view of the IFNγ-biased response within the vaccinated-challenged host.

In summary, our data suggests that animals which have been vaccinated with an adjuvanted, inactivated mutant strain of *E. coli* O157:H7, showed an increase in γδ T cells at the follicular RAJ. Within the tissue from adjuvanted-vaccinated animals, lymphocytes express IFNγ to a greater extent than non-vaccinated or non-adjuvanted animals. Furthermore, the transcriptional profile at the follicular RAJ shows characteristic differences between the experimental groups, suggesting that adjuvanted-vaccine will promote an inflammatory-skewing cellular profile. Although the IFNγ-specific effects on the RAJ are unknown, treating epithelial cells with IFNγ in vitro reduced O157 adherence which suggested that vaccination elicited IFNγ could play a role in the reduction of O157 fecal shedding. The consideration of vaccine-driven cell-mediated IFNγ at the tissue site could have a significant impact on the future development of O157 directed vaccinations.

## Materials and methods

### Animals

Clinically healthy Jersey male cattle (n = 12) of approximately 6 months of age that were used for vaccination and *E. coli* O157:H7 (O157) challenge in a previously published study^18^ served as a source of blood, fecal, and tissues samples for the current study. All animals were challenges with *E. coli* O157:H7, NoAdj-NoVac group served as an unvaccinated control, NoAdj-Vac (no adjuvant containing vaccine) and Adj-Vac (adjuvanted vaccine) groups received their respective treatments prior to vaccination (see additional details in following section). Animals were housed in BSL-2 conditions and fed a maintaining diet of grain. Animals were randomly separated and isolated into groups of 4 and allowed to condition to their environment for 2 weeks prior to start of experiment. Animal procedures employed in these studies were approved by the National Animal Disease Center Institutional Animal Care and Use Committee (IACUC) and performed in accordance with the relevant IACUC and agency guidelines and regulations.

### Generation of O157 vaccine strain, vaccination, and challenge procedures

O157 vaccine strain (*E. coli* O157:H7 Δ*hha*, Strain NADC 6597) was generated as described previously^11,45,46^. Formaldehyde inactivation of the vaccine strain and use of the inactivated strain in the preparation of vaccine formulations have also been described previously^12^. Briefly, vaccine formulations contained 1.2 mL of phosphate buffered saline (PBS) containing 10^9^ inactivated bacterial cells of the vaccine strain mixed with either a 0.8 mL of PBS (Non-adjuvanted-vaccine, abbreviated as NoAdj-Vac) or 0/8 mL of Seppic Montanide ISA 61 VG adjuvant (Adjuvanted-vaccine, abbreviated as Adj-Vac). PBS only injected animals were utilized as control (NoAdj-NoVac). Animals were prime-vaccinated for three weeks prior to vaccine-boost. Three weeks after vaccine-boost all animals were orally inoculated with *E. coli* O157:H7 strain NADC 6564^12^. Animals were monitored after vaccination and boost for adverse site reactions. Three of the twelve animals displayed a small site reaction in response to boost. Animals were monitored for four additional weeks after challenge and were humanely euthanized for collection of tissues.

### Tissues for microscopic analysis

Following euthanasia, sections of rectoanal junction (RAJ) were collected and tissues collected into follicular and squamous region of the RAJ. The follicular and squamous adjacent RAJ regions were identified visually, as tissue morphology is indicative of these rectal sections. Tissues were fixed by immersion in 10% neutral buffered formalin for 24 h, and then transferred to 70% ethanol. Sections were processed by standard paraffin-embedment techniques, cut in 5 μm sections, and stained with hematoxylin and eosin (HE) or sectioned into 20 μm sections for RNA isolation. Duplicate sections of follicular or squamous RAJ tissue were embedded into optimal cutting temperature (OCT) compound and flash frozen in dry-ice/ethanol bath. Two-micron sections were mounted onto positively charged slides, air dried for 10 min, and excess OCT was removed. Next, sections were fixed and permeabilized with acetone/ethanol (50:50) solution, blocked with StartingBlock reagent (Thermo Scientific), and stored at 4 ℃. Formalin fixed sections were used for microscopic analysis of lymphoid structures and in situ hybridization or RNA isolation, and frozen OCT sections used for immunofluorescent labeling.

### Immunofluorescent labeling (IFA) and imaging of slides

OCT sectioned and blocked slides were washed 3 × with phosphate buffered saline (PBS, Thermo Scientific-Gibco). Tissue sections on the slides were contoured with a hydrophobic barrier (PAP Pen, Vector Labs, Burlington Ontario, Canada) and a stained for IFNγ [1:50] (Clone 7B6, IgG1, Bio-Rad) and γδTCR [1:100] (Clone GB21A, IgG2b, Washington State University). Slides were allowed to incubate in a humidified light-blocking chamber for 1 h, washed 3 × with PBS, and incubated with anti-murine IgG1-FITC [1:500] or anti-murine IgG2b-PE [1:1000] (all secondaries from Southern Biotech) and allowed to incubate in a humidified light-blocking chamber for 1 h. Slides were washed 3 × and mounted with nuclear stain (DAPI) containing medium (VectorLabs). Secondary only sections were included for background fluorescence. Slides were allowed to cure for 24 h and were imaged using a Nikon camera and Leica microscope setup. Secondary only sections were used to adjust camera settings to set signal baseline. Triplicate slides were created per tissue block (animal) and three images were collected within each slide. Data is represented as average of three slides per animal. Images were analyzed using ImageJ software (NIH, Bethesda MD) for color intensity.

### Detection of gene transcript in tissues

Cytokine mRNA transcripts were visualized using in situ hybridization (ISH) methods that have been described previously^47–49^. *Bos taurus*-specific proprietary probe combinations for IFNG (Cat #315581), IL10 (Cat #420941) or TRDC (Cat #407481) for lymphocyte subset γδ were detected according to the manufacturer’s instructions for RNAScope 2.0 (Advanced Cell Diagnostics, Hayward, CA, USA). Briefly, sections 5-µm thick, cut from formalin-fixed, paraffin-embedded tissues were heated for 60 min at 60 °C in a HybEZ ™ hybridization oven (Advanced Cell Diagnostics). Tissues were deparaffinized in xylene followed by rehydration in an ethanol series and air-dried for 5 min. Tissue sections were incubated with pretreatment 1 solution (endogenous peroxidase block) for 10 min at room temperature (RT). Slides were rinsed by immersion in double-distilled water (ddH2O), followed by immersion in pretreatment 2 (antigen retrieval citrate buffer) for 15 min at 100–104 °C (boiling). Slides were washed in ddH2O and pretreatment 3 (protease) was applied for 30 min at 40 °C. Slides were washed in ddH2O and target or control probes applied with incubation at 40 °C for 2 h followed by a rinse in a wash buffer (Advanced Cell Diagnostics) for 2 min at RT. Signal amplification reagents 1 through 6 were serially applied for 30 min, 15 min, 30 min, 15 min, 30 min and 15 min, respectively. Slides were rinsed in wash buffer for 2 min between amplification reagents. Incubations with amplifier reagents 1 through 4 were done at 40 °C, while incubations with amplifier reagents 5 and 6 were done at RT. Positive signal was visualized using Fast Red chromogenic substrate and a Gill’s hematoxylin counterstain. Slides were then dehydrated through an ethanol series to xylene. After drying 15 min at 60 °C, slides were coverslipped using mounting media (EcoMount, Biocare Medical, Concord, CA, USA). The positive control probe consisted of a proprietary probe for *Bos taurus* cyclophilin B (Cat # 3194510), while the negative control probe targeted *dapB* of *Bacillus subtilis* (Cat # 312038).

### Morphometry

Tissue sections after H&E, HC and ISH assays were scanned at 40X maximum magnification and digitized using the Aperio ScanScope XT workstation (Aperio Technology, Inc., Vista, CA, USA) and Aperio eSlide Manager version is 12.3.2.8013 (Leica Biosystems, Inc, Buffalo Grove, IL; https://www.leicabiosystems.com). Digitized images were analyzed using image analysis software HALO version 3.1, ISH-module version 3.0.3 (Indica Labs, Inc., Corrales, NM; https://www.indicalab.com/halo/). For H&E stained microscopy images, areas (2.0 µm) of GALT were outlined and the number of lymphocytes within GALT (identified by the software) was normalized by dividing by the length of mucosa for each individual section to achieve the number of lymphocytes within GALT per unit length of mucosa. Although measuring follicular cells in the RAJ is difficult, the authors feel that using GALT cells per mucosal length is a logical and meaningful parameter. Using the length of mucosa or mucosal structures such as the muscularis mucosa is a technique for which there is a precedence^50,51^. Similarly, for ISH, image analysis software identified cells labeled for lymphocyte subsets (TRD, CD4 or CD8A) or cytokine mRNA (IL10 or IFN gamma) and the number of cells per unit length of the mucosa calculated.

### RNA extraction and RT-qPCR

Formalin-fixed paraffin embedded tissues were sectioned into 20-micron sections and placed into sterilized, nuclease free tubes. Sections were treated and RNA isolated according to manufacturer’s instruction (RecoverAll, Thermo Scientific-Ambion). Recovered RNA was analyzed for integrity and quantified using a Bioanalyzer and RNA 6000 Nano Kit (Agilent). Amounts of RNA were diluted into 500 ng/µL using nuclease-free water (Thermo Scientific). Gene analysis was performed utilizing RT2 profiling array for bovine inflammatory genes per manufacturer’s instruction (Inflammatory Cytokines and Receptors Array Plate, Qiagen). Briefly, 500 ng of RNA (1 µL), 1 µL of nuclease free water (Thermo Scientific) and 18 µL of 1-step RT-qPCR reaction buffer (OneStep Kit, Qiagen) was added to each well. A BioRad CFX96 Real Time PCR Systems thermocycler (Bio-Rad, Hercules, CA) was used. Amplification conditions for were: 30 min 50 °C, 10 min at 95 °C, 40 cycles of 15 s 95 °C and 1 min 60 °C (measure florescence step) and a dissociation step of 15 s 95 °C, 1 min 60 °C, 15 s 95 °C, 15 s 60 °C. Ct values were calculated and normalized to the endogenous control and expressed relative to No-Adj/No-Vac tissue sections using the 2^−ΔΔCT^ method.

### ELISPOT and cell isolation

Messenteric lymph node cells and splenocytes were isolated as previously described^52^. Briefly, tissue sections were homogenized utilizing a gentleMACS Octo Dissociator with M-tubes (Miltenyi Biotec). Cell homogenate was lysed for RBCs, washed with PBS, strained using a 40 µm nylon strainer, counted for viable cells and resuspended in cRPMI. ELISPOTs were performed in accordance with manufacturer’s instructions (Bovine IFN ELISPOT Kit, R&D Systems). Lysates for stimulating cells were collected as described previously^18^. Briefly, 10^5^ lymph node cells or splenocytes were seeded into each well of ELISpot plate and stimulated with either 1 ug of Shiga toxin *E. coli* lysate, 1 µg of vaccine *E. coli* lysate (NADC 6564), or media only. Plates were incubated for 48 h at 37 °C, washed, and spots developed with anti-bovine IFNγ. Plates were imaged and spots enumerate with an ImmunoSpot analyzer (Cellular Technology Limited, Cleveland, OH).

### O157 adherence to HEp-2 and bovine ilial epithelial cells (BIECs)

HEp-2 cells were seeded at 5 × 10^5^ cells/well, grown as described previously^18,53^ to confluency, and either untreated or treated with recombinant bovine (rb) interferon-γ (R&D Systems). After 60 min of incubation at 37 °C, 10^9^ CFU/mL of O157, grown overnight in LB broth at 37 °C and 200 rpm of shaking, was added per well. Plates were incubated at 37 °C for 3 h. Non-adhered O157 was washed away using PBS to leave any strongly adhered O157 to the epithelial cell layer. Adhered HEp-2 cells were gently lysed in 1% Triton-X100 to release O157. Lysate-containing whole cell O157 samples were diluted and plated on LB agar plates. After incubation at 37 °C for 24 h colonies that grew on these plates were counted. For determining O157 adherence to BIECs (kindly provided by Dr. Radhey Kaushik, South Dakota State University), confluent BIEC cells, grown as described previously^31,54^, were treated with media alone or rbIFNγ for approximately 16 h at 39 °C. Approximately 10^7^ O157 cells were added to wells for 2 h, and non-adhered O157 bacteria were washed away with PBS. Attached O157 bacterial cells were released by addition of 0.5% Triton-X in PBS for 5–10 min. The cell lysate was serially-diluted, plated and bacterial counts were determined by counting number of colonies that grew on plates as described above. The data is expressed relative to average attached O157 in media-only wells. Each assay included 3 wells with each treatment and data includes two separate experiments.

### Statistical analysis

Data were analyzed with GraphPad Prism7 (GraphPad Software, San Diego, CA; https://www.graphpad.com/company/). Except where noted in the figure legend, statistical analysis performed utilizing one way-ANOVA with multiple comparison of means. *p < 0.05, **p < 0.01, ***p < 0.001.

### Ethics statement

Animal studies were approved by the Institutional Animal Care and Use Committee (IACUC) at the National Animal Disease Center. The study was carried out in compliance with the ARRIVE guidelines.

## Supplementary Information

Supplementary Information 1.

Supplementary Information 2.
