# Supplementary material for: Mucosal IFNγ production and potential role in protection in Escherichia coli O157:H7 vaccinated and challenged cattle
Source: Sci Rep. 2021 May 7;11:9769. doi: 10.1038/s41598-021-89113-7 (PMC8105325; doi:10.1038/s41598-021-89113-7)
Supplement: Supplementary file 1 — Supplementary Information 1. [file 41598_2021_89113_MOESM1_ESM.docx]

**Mucosal IFN-γ production and potential role in protection in *Escherichia coli* O157:H7 vaccinated and challenged cattle**

Robert G. Schaut^1,2,3,a^, Mitchell V. Palmer^1,4^ , Paola M. Boggiatto^1,4^, Indira T. Kudva^1,2^, Crystal L. Loving^1,2^ ,Vijay K. Sharma^1,2,*^


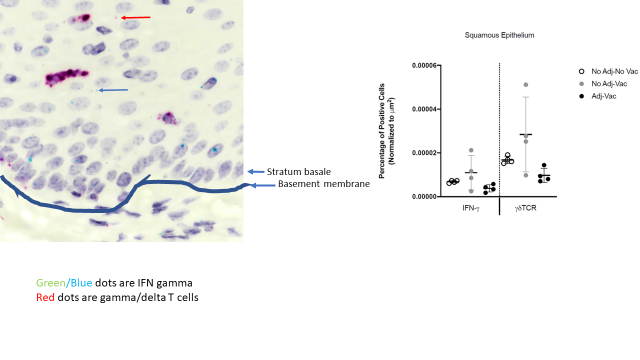


**Supplementary Figure S1. Detection of IFN-γ and γδ T cells transcripts in squamous region of RAJ by RNA in situ hybridization.** Squamous region of recto-anal junction (RAJ) was isolated from a representative animal which received no adjuvant and no vaccine strain (NoAdj-NoVac), received non-adjuvanted-Δ*hha*-*E. coli* vaccine (NoAdj-Vac) , or received adjuvanted-Δ*hha*-*E. coli* vaccine (Adj-Vac). Figure A represents section of squamous epithelium of animal which received adjuvanted-Δ*hha*-*E. coli* vaccine (Adj-Vac). Red arrows indicate γδ T cell staining and blue arrows indicate IFNγ staining. Graph on the right shows percentage of positive γδ TCR and IFNγ. Open circles indicate NoAdj-NoVac, grey circles represent NoAdj-Vac, and black circles represent Adj-Vac group. Each symbol represents an individual animal. N=4 per group. Bars = +/- SD. One-way ANOVA with Tukey’s post-test was utilized for statistical analysis. **** *p*<0.001, * *p*<0.05.

**
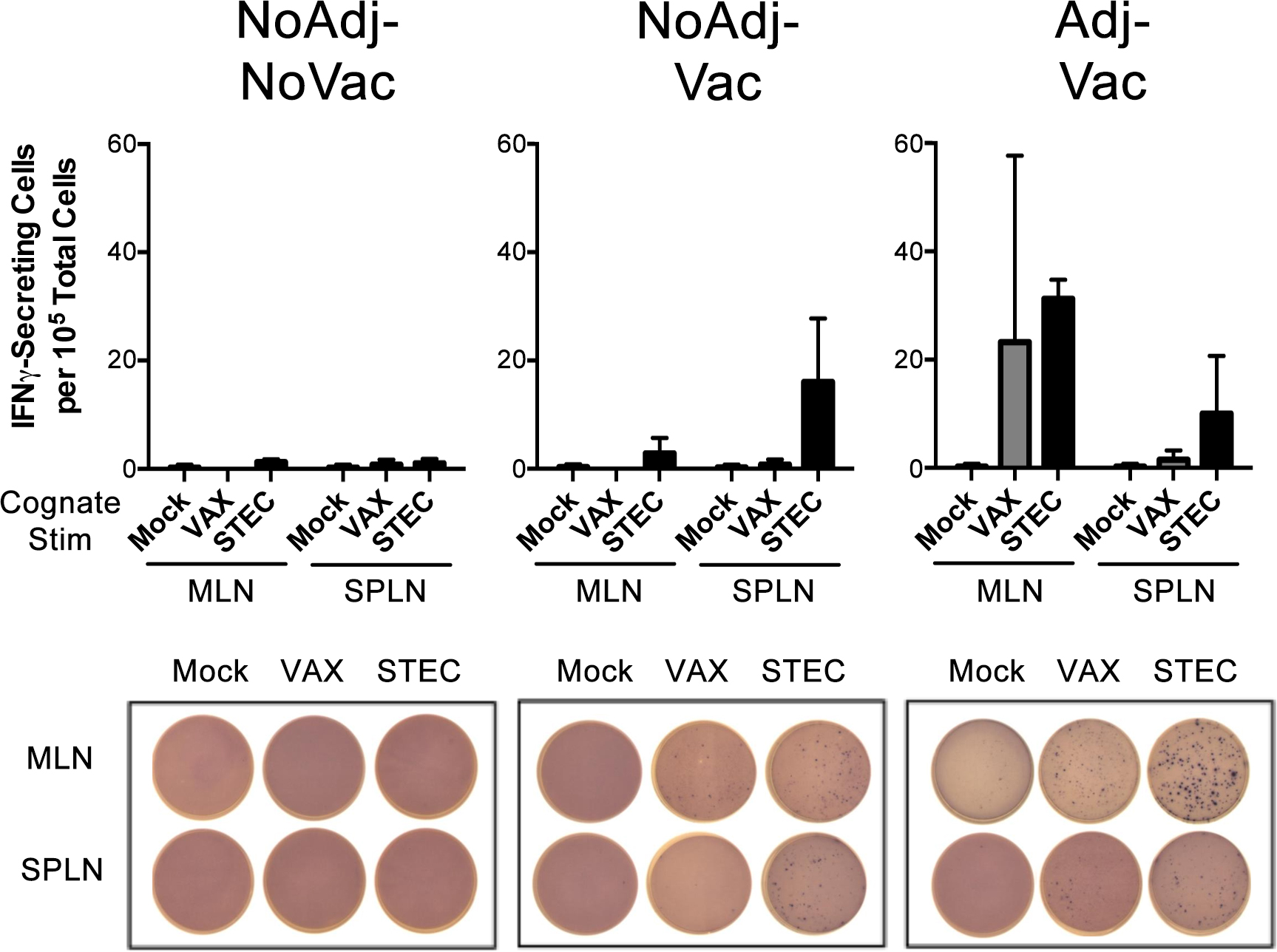
**

**Supplementary Figure S2. Vaccination induced both a localized and systemic inflammatory response to *E. coli* lysate simulation.** Cells isolated from the mesenteric lymph node (MLN) and spleen (SPLN) were assayed for IFNγ producing cells by ELISPOT. A) The # of IFN-γ secreting cells detected in respective tissues from Non-adjuvanted-non-vaccinated (NoAdj-NoVac) (left panel), Non-adjuvanted-Δ*hha* *E. coli* vaccinated (NoAdj-Vac) (middle panel) or adjuvanted- Δ*hha* *E. coli* vaccinated (Adj-Vac) (right panel) animals. Open bars represent medium-only, no stimulation controls (mock), grey bars represent Δ*hha* *E. coli* lysate stimulation (VAX), and black bars represent STEC challenge lysate simulation (STEC). Cells were plated at 10^5^ cells/well and stimulated for 24 h. B) Representative images of spots in ELISpot wells for NoAdj-NoVac (left), NoAdj-Vac (middle) or Adj-Vac (right). MLN = mesenteric lymph node-lymphocytes, SPLN = splenocytes, Mock = cell culture medium only stimulation, VAX = Δ*hha* *E. coli* lysate stimulation, STEC = shiga-toxin *E. coli* lymphocyte stimulation. All error bars indicate SD, n=4 per group. * p<0.05, **p<0.01, ***p<0.001 by one-way ANOVA with Tukey’s post-test.
